# O1-6 Objectively measured physical activity levels and association with BMI z-score among children and adolescents in Morocco

**DOI:** 10.1093/eurpub/ckac094.006

**Published:** 2022-08-29

**Authors:** Issad Baddou, Imane El Harchaoui, Kaoutar Benjeddou, Imane El Menchawy, Naima Saeid, Khalid El Kari, Mohammed Elmzibri, Asmaa El Hamdouchi, Hassan Aguenaou

**Affiliations:** Unité Mixte de Recherche Nutrition et Alimentation CNESTEN- Ibn Tofail University (URAC39), Regional Designated Center of Nutrition Associated with AFRA/IAEA. Rabat, Morocco

**Keywords:** BMI z-score, physical activity, accelerometer, children, adolescent

## Abstract

**Background:**

Physical activity is associated with improved psychological well-being and lower levels of cardio metabolic risk factors, diabetes, obesity among children and adolescent. The purpose of the study was 1- to examine gender, type of day, and age grade differences in objectively PA; 2- to examine the attainment of recommended physical activity guidelines; 3-to examine the association between PA levels and BMI z-score among children and adolescents in Morocco.

**Method:**

172 Moroccan children/adolescents (mean age = 10.92 ± 1.55 years, mean BMI z-score = -0.16 ± 1.33; 19.2 % overweight) were recruited for this study and wore a tri-axial accelerometer (GT3X+) for 7 consecutive days. Two-way analysis of covariance was used to examine gender and age grade differences in physical activity level separately for weekdays and weekends, adjusted for body mass index for age (BMI z-score) and wear time. Logistic regression analyses were conducted to examine independent relationships between attainments of physical activity guidelines and gender. Pearson correlation was used to assess the association between physical activity levels and BMI z-score.

**Results:**

In both weekends and weekday, children spent more time in Light physical activity than adolescents (p > 0.001), boys were more engaged in moderate activity (p > 0.001) and vigorous (p > 0.001) activity and took more steps than girls. Boys were eight time more likely to meet the recommendation for at least 60 min of moderate to vigorous physical activity per day than girls (OR: 8.569; 95% [CI]: 4.23-17.32), p > 0.001. Among adolescent, moderate to vigorous PA were inversely correlated with BMI z-score (r = -0,213; p = 0.04).

**Conclusion:**

The findings can shed light on the need of urgent scaling up of implementation of known effective policies and programmes for adolescents and girls to increase their involvement in PA.

*Acknowledgements*

This study was performed with the support of the International Atomic Energy Agency (CRP E4.30.24; RAF 6042).

